# Status of bone health and association of socio-demographic characteristics with Bone Mineral Density in Pakistani Females

**DOI:** 10.12669/pjms.35.3.551

**Published:** 2019

**Authors:** Saba Tariq, Mukhtiar Baig, Sundus Tariq, Muhammad Shahzad

**Affiliations:** 1 *Saba Tariq, MBBS, M.Phil. Associate Professor of Pharmacology, Research Scholar (Pharmacology), University of Health Sciences, Lahore, Pakistan., University Medical & Dental College, Faisalabad- 38000, Pakistan*; 2 *Prof. Mukhtiar Baig, MBBS, M.Phil, PhD. Department of Clinical Biochemistry, Faculty of Medicine, Rabigh, King Abdulaziz University, Jeddah- 21589, KSA*; 3 *Sundus Tariq, MBBS, M.Phil. Associate Professor of Physiology, Research Scholar (Physiology), University of Health Sciences, Lahore, Pakistan., University Medical & Dental College, Faisalabad- 38000, Pakistan*; 4 *Muhammad Shahzad, M.Phil, PhD. Associate Professor of Pharmacology, University of Health Sciences, Lahore, Pakistan*

**Keywords:** BMD, osteoporosis prevalence, premenopausal, postmenopausal, Pakistani females

## Abstract

**Background & Objective:**

The “silent thief” of bone osteoporosis is associated with various modifiable factors, identifying these factors is important in decreasing the prevalence of this highly prevalent disease. Therefore, this study was planned to identify these risk factors for osteoporosis in premenopausal and postmenopausal Pakistani women.

**Methods:**

A total of 1205 pre and postmenopausal females between the ages of 20 to 80 years were selected. Detailed history about the socio-demographic characteristics including age, education, profession, marital and resident status was recorded. Medical and gynecological history was also taken after informed consent Bone health of females was assessed using calcaneal ultrasound bone densitometer. SPSS 22.0 was used to analyze data.

**Results:**

Univariate analysis showed that age (30-39 yrs, and 60-69 yrs), occupation (housewives) and education (secondary and primary education, illiterate) were significantly associated with low bone mass density (LBMD). Multivariate analysis showed that age 30-39 years (OR=0.25 95%CI 0.13 – 0.49), age 40-49 years (OR=0.30 95%CI 0.15 – 0.59), age 50-59 years (OR=0.42 95%CI 0.22 – 0.79), primary education (OR=3.83, 95%CI 2.30 - 6.38) and illiteracy (OR=3.83 95%CI 2.52 – 5.82), were significantly associated with LBMD. The prevalence of osteopenia and osteoporosis was 29.8%, 27.2%, respectively, while 43% subjects had normal BMD.

**Conclusion:**

It is concluded that, within Pakistani population, the prevalence of osteopenia is high even at an early age group and the odds of having LBMD are more in less educated or illiterate women.

## INTRODUCTION

Osteoporosis (OP) is a multi-factorial problem and recognition of modifiable factors is crucial for healthy aging and to cut down the medical, social and personal costs of fracture.^1^Moreover, patients with any osteoporosis-related fracture could have unhealthy state of mind and consequently have psychological symptoms such as depression and low self-esteem, that is, because of their physical limitations, changed lifestyle and pain in the fracture.^2^ Fractures among postmenopausal women have an enormous economic impact and high financial burden on health system due to increasing utilization of health resources, hospitalization, nursing home requirements, loss of productivity and reduced mobility after hip fractures.^3^ In Pakistan, the reported prevalence of osteopenia is 34%-72.9%, and OP is 2.4%-30.90%.^4^ Makhdoom et al. (2014), found that 30.9% and 45.60% Pakistani females were osteoporotic and osteopenic, respectively.^5^ In 2009, International Osteoporosis Foundation (IOF) reported that in Pakistan, 7.2 million women have OP out of a total estimated 9.9 million subjects. Additionally, it is anticipated that incidence of osteopenia is about 40 million in Pakistanis, and both genders are equally suffering from this problem. It is also estimated that the prevalence of OP in Pakistan would increase to 11.3 million (2020) and 12.91 million (2050).^6^

Recently, a community-based survey from Saudi Arabia revealed that 53% women had low BMD,^7^ and another study in South Korea reported the prevalence of OP as 32.3% and osteopenia as 49.9 % in female population.^8^

The literature shows that marital status, educational levels, nature of job, place of residence have a significant impact on the bone mass density and risk of fractures. These factors need to be given proper importance while assessing the patients for OP and fractures. Numerous literature is available regarding the role of education,^7^,^9^ marital status and occupation,^10^ and these are related to the varying prevalence of OP and fracture rates.

In Pakistan, there is a scarcity of data on epidemiology and demographics of OP and associated fractures. It is imperative to identify the essential risk factors for OP. Limited information is available regarding the effect of age, marital status, educational levels, occupation, and place of living on bone health in our part of the world. Therefore, this study was planned to measure the bone mineral density in pre- and postmenopausal Pakistani women and to identify associated risk factors for osteoporosis.

## METHODS

The present cross-sectional, exploratory study was conducted at the Orthopaedic Department, Shaikh Zayed Hospital, Lahore, Pakistan. The calculated sample size was 380, and it was calculated in health studies version 2.0.21 World Health Organization, by the following formula keeping the confidence level equal to 95%, the margin of error equal to 5% and anticipated proportion of osteoporosis (30%).^5^


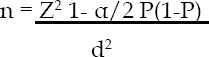


**Fig.1:**
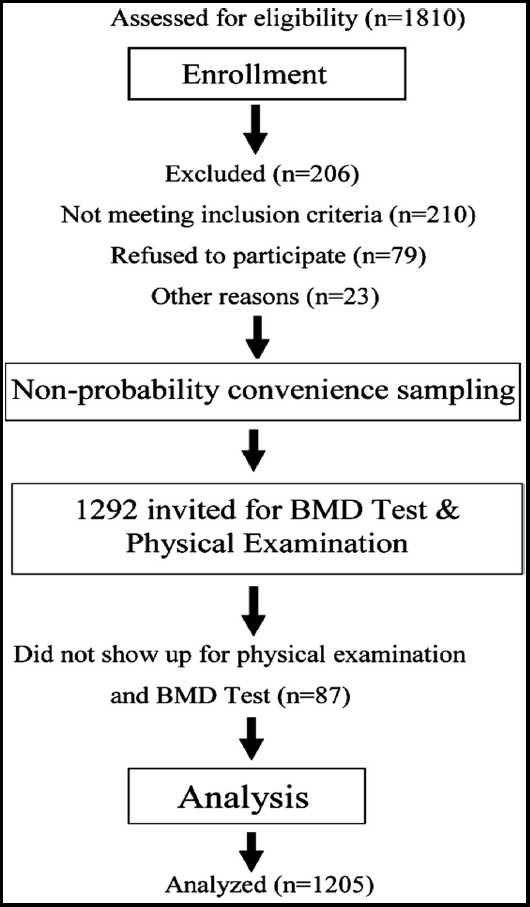
Study subjects selection chart.

All the participants were selected by convenience sampling method. A written informed consent was taken from all subjects. Females of age group 20-80 years were enrolled, and 1810 females were interviewed for participation in this study. Many of them did not fulfill the inclusion criteria, so those females were excluded, and finally, 1205 women participated in the study (Fig.1). Information was collected on a specially designed proforma. The detail of the inclusion and exclusion criteria is mentioned in our other study.^11^

Peripheral ultrasound bone densitometer (Sonsot 3000, manufactured by OsteoSys Co., Ltd. Seoul, Korea) was used for the assessment of BMD on the calcaneus (heel). Research indicates that Quantitative Ultrasound (QUS) may be as accurate as Dual-energy X-ray Absorptiometry (DEXA) in predicting the risk of fracture and diagnosing osteoporosis.^12^,^13^

World Health Organization (WHO) criteria for diagnosing OP and osteopenia were used: t-score < -2.5 were considered as OP; t-score -1 to -2.5 as osteopenia, and those with t-scores > -1were considered as normal.^14^

### Statistical Analysis

The data were analyzed using SPSS (Statistical Package for Social Sciences) version 22. Mean ± SD was given for quantitative variables. Shapiro-Wilk test was used to check the normality of data. Independent sample t-test and chi-square (χ^2^) test was used for comparisons. By dividing our subjects into two categories, *i.e*., low bone mineral density (LBMD) and normal bone mineral density (NBMD), univariate logistic regression was applied to determine the influence of marital status, profession, residence, educational levels and age factors associated with bone mineral density. A multivariate logistic regression analysis was conducted, and adjusted odds ratios and the associated 95% confidence intervals were computed. The significance of p-value was considered at < 0.05.

## RESULTS

A total of 1810 women were initially assessed for eligibility for this study, 206 were excluded because of taking antiresorptive treatment, 210 did not meet inclusion criteria, 79 refused to participate, 23 were excluded for other reasons. Finally, selected women were invited for BMD test, and physical examination but 87 did not show up (Fig.1). Baseline characteristics of study group is given in Table-I.

**Table-I:** General characteristics of the participants overall and comparison according to menopausal status.

Parameters	Overall	Premenopausal (a)	Postmenopausal (b)	p-value
	N=1205 N(%)	N= 487 N(%)	N=718 N(%)	(a vs b)
***Marital status***				
Married	1038 (86.1%)	376 (36.2%)	662 (63.8%)	< 0.001
Unmarried	167 (13.9%)	111 (66.5%)	56 (33.5%)	
Age of menarche (mean±SD, years)	-	14.9±1.4	15.2±1.3	<0.001
Year since menopause (mean±SD, years)	-	-	10.2±7.3	
Age of menopause (mean±SD, years)	-	-	48.53±2.57	
Age (mean±SD)	49.9±12.5	37.3 ± 8.4	58.4 ± 6.0	< 0.001
***Occupation***				
House wife	983 (81.6%)	337 (34.3%)	646 (65.7%)	< 0.001
Private job	222 (18.4%)	150 (67.6%)	72 (32.4%)	
***BMD***				
Normal	518 (43.0%)	282 (54.4%)	236 (45.6%)	< 0.001
Osteopenic	359 (29.8%)	157 (43.7%)	202 (56.3%)	
Osteoporotic	328 (27.2%)	48 (14.6%)	280 (85.4%)	
***Educational level***				
Graduate	158 (13.1%)	96 (60.8%)	62 (39.2%)	< 0.001
Higher secondary	101 (8.4%)	53 (52.5%)	48 (47.5%)	
Secondary	178 (14.8%)	68 (38.2%)	110 (61.8%)	
Primary	208 (17.3%)	48 (23%)	160 (77%)	
Illiterate	560 (46.5%)	222 (39.6%)	338 (60.4%)	
***Living place (residence)***				
Urban	674 (55.9%)	276 (41%)	398 (59%)	0.670
Rural	531 (44.1%)	211 (39.7%)	320 (60.3%)	

There was a significant difference in number of osteoporotic, osteopenic and normal subjects in married and unmarried subjects (p<0.001), in housewives and working women (p<0.002), at all educational levels (p<0.001), in age groups (p<0.001), but no difference was found in living place (urban and rural) (0.321) (Table-II).

**Table-II:** Comparison of normal, osteopenic and osteoporotic subjects according to marital status, occupation, educational level, place of living (residence) and age.

Variable	Normal (N=518)	Osteopenic (N=359)	Osteoporotic (N=328)	P-value
***Marital status***				
Married	442(42.6)	292 (28.1)	304(29.3)	<0.001
Unmarried	76(45.5)	67(40.1)	24(14.4)	
***Occupation***				
House wife	400(40.7)	299(30.4)	284(28.9)	0.002
Private job	118(53.2)	60(27)	44(19.8)	
***Educational level***				
Illiterate	218(38.9)	172(30.7)	170(30.4)	<0.001
Primary	58(27.9)	72(34.6)	78(37.5)	
Secondary	86(48.3)	52(29.2)	40(22.5)	
Higher secondary	54(53.5)	29(28.7)	18(17.8)	
Graduate	102(64.6)	34(21.5)	22(13.9)	
***Residence***				
Urban	288(42.7)	192(28.5)	194(28.8)	0.321
Rural	230(43.3)	167(31.5)	134(25.2)	
***Age***				
20-29yr	54(44.6)	63(52.1)	4(3.3)	<0.001
30-39	104(77.6)	14(10.4)	16(11.9)	
40-49	124(53.4)	80(34.5)	28(12.1)	
50-59	162(40.3)	110(27.4)	130(32.3)	
60-69	66(22.9)	78(27.1)	144(50)	
70-85	8(28.6)	14(50)	6(21.4)	

Univariate analysis showed that age (30-39 years, and 60-69 years), occupation (housewives) and education (secondary and primary education, illiterate), were significantly associated with LBMD. (Table-III). Multivariate analysis showed that age 30-39 years (OR=0.25, 95%CI 0.13 – 0.49), age 40-49 years (OR=0.30, 95%CI 0.15 - 0.59), age 50-59 years (OR=0.42, 95%CI 0.22 – 0.79), primary education (OR=3.83, 95%CI 2.30 - 6.38) and illiteracy (OR=3.83, 95%CI 2.52 – 5.82) were significantly associated with LBMD. Marital status and living place were not significantly associated with LBMD on univariate or multivariate analysis (Table-III).

**Table-III:** Univariate and multivariate logistic regression analysis showing odds ratio between educational level, age, marital status, profession and place of living (residence) with LBMD.

Variable	Univariate analysis		Multivariate analysis	

	Odds ratio (95% CI)	P-value	Odds ratio (95% CI)	P-value
***Educational level***				
Graduate	Reference		Reference	
Higher secondary	0.95 (0.57 – 1.56) 0.945		0.70 (0.41 – 1.21)	0.202
Secondary	1.82 (1.17 – 2.83) 0.008		1.52 (0.94 – 2.44)	0.085
Primary	4.43 (2.75 – 7.13) <0.001		3.83 (2.30 - 6.38)	<0.001
Illiterate	3.47 (2.39 – 5.04) <0.001		3.83 (2.52 – 5.82)	<0.001
***Age***				
20-29 yr	Reference		Reference	
30-39	0.38 (0.23 – 0.65) <0.001		0.25 (0.13 – 0.49)	<0.001
40-49	0.63 (0.38 – 1.03) 0.063		0.30 (0.15 – 0.59)	<0.001
50-59	0.76 (0.48 – 1.20) 0.239		0.42 (0.22 – 0.79)	0.007
60-69	1.93 (1.14 – 3.27) 0.014		1.04 (0.53 – 2.04)	0.920
70-85	1.98 (0.64 – 6.16) 0.239		0.67 (0.19 – 2.35)	0.536
***Marital status***				
Unmarried	Reference		Reference	
Married	0.83 (0.57 – 1.21) 0.343		0.73 (0.43 – 1.24)	0.243
***Profession***				
Job	Reference		Reference	
HW	1.42 (1.04 – 1.94) 0.028		1.17 (0.79 – 1.78)	0.445
***Living place (residence)***				
Rural	Reference		Reference	
Urban	1.22 (0.95 – 1.57) 0.117		1.11 (0.84 – 1.45)	0.471

HW=housewives.

## DISCUSSION

In the present study, the prevalence of osteopenia and osteoporosis was 29.8%, 27.2%, respectively. These results showed that a large number of the apparently healthy population have low BMD values as they suffer from both osteopenia and osteoporosis. Alarmingly, a large proportion of premenopausal women have low BMD. The present study results are consistent with a number of studies that have found low BMD in Pakistani population.^4^,^5^,^15^

The percentage of osteoporotic was higher and osteopenic was lower in our study as compared to another study that reported 64.1% of the participants were osteopenic and 18.6% were osteoporotic within Pakistani population.^16^ The percentage of osteoporotic subjects is double in our study compared to another study that demonstrated 12.9% prevalence of osteoporosis.^15^ However, one reason for the difference could be the time of the study, which was conducted five years ago, and now, the prevalence has increased. A similar study, reported low BMD at all skeletal sites among low socioeconomic women in India, in that study half of the participants were osteopenic, and one-third were osteoporotic, and it was suggested that insufficient nutrition was the major contributing factor.^17^ A more recent study among postmenopausal females found that 42.5% of females were osteoporotic, and 44.9% were osteopenic.^18^

Several studies have found that calcium and vitamin D deficiency is prevalent in Pakistani population. Vitamin D deficiency in early age badly affects peak bone mass in grown-ups and could augment the osteoporosis risk.^19^,^20^ An IFO report by Mithal et al. (2014), suggested that there could be multiple causes of the high occurrence of vitamin D deficiency like less exposure to sun, low intake of vitamin D, inadequate food fortification with vitamin D, environmental pollution, customary dress wearing, and pigmented skin.^21^

A study reported that generally, the daily intake of calcium in adult Pakistanis is low. It was 400-600 mg/day while the recommended daily allowance is 1000-1200 mg.^22^,^23^ Therefore, we should develop strategies to increase calcium intake in our population.

Our results clearly show that with an increase in the level of education, there are more chances of good bone health and BMD. Our results are similar to another study that reported an inverse relationship between prevalence of OP and educational level. An increase in educational level was related to a considerably decreased risk for OP.^24^ In multiple logistic regressions analysis, levels of education showed a predictive role toward LBMD. Education plays an important protective role against the prevalence of osteoporosis and non-educated and less educated women have more chances of having low BMD. There are several studies found in the literature regarding the influence of education on BMD and fracture.^10^,^24^,^25^

The present study found that married women had more chances of developing osteoporosis as compared to unmarried women. Recently, a study in Poland stated specific function of marital condition in both skeletal status and hormone replacement therapy (HRT) usage. They noted borderline significant differences about fracture occurrence, but after adjusting age, these differences became insignificant.^9^ There could be several contributing factors for the higher occurrence of osteopenia and osteoporosis among married females like increasing age, multiparty, lactation, insufficient diet and others.^26^

Our study subjects were divided into six age categories. It has been observed that in our population, osteopenia occurs at a very early age; that is why most women develop OP within ten years of their menopause, and a large number of the women consequently develop osteoporotic fractures. A study reported a significantly negative correlation between age and BMD whereas weight and BMI have a positive impact on BMD.^27^

The striking finding of the current study was that women ages 20-29 had lower BMD than those ages 30-59. It seems that particular cohort of young women exposed to a period of nutritional insufficiency during a critical developmental period due to which they suffer from LBMD at an early age. Similarly, some other studies have mentioned that age, education, and dietary products are associated with low BMD.^20^-^25^,^28^,^29^

In the present study, it was observed OP is more common in housewives as compared to women who were doing some job. Researchers reported that femoral neck and total hip BMD values were related with the occupational character. They further revealed that that the skeletal status was considerably healthier with women who stand while working in comparison to those who sit while working, but both did not affect the incidence of fractures.^4^ In multiple logistic regression analysis, the profession was not found as a predictive factor against BMD.

The present study results advocate that level of education, nature of the occupation, matrimonial status, and living place should be taken into consideration in OP management projects and OP prevention health programs.

### Limitation of the study

The main limitation of this study was that we did not measure BMD by DEXA because it is expensive and has radiation hazards.

## CONCLUSION

The low bone mass was prevalent and overall bone status of our study participants was not good. Additionally, It is concluded that, within Pakistani population, the prevalence of osteopenia is high even at an early age group and the odds of having LBMD are more in less educated or illiterate women.
